# Is there a role for bradykinin in cerebral malaria pathogenesis?

**DOI:** 10.3389/fcimb.2023.1184896

**Published:** 2023-08-10

**Authors:** Alessandro de Sa Pinheiro, James W. Kazura, Ana Acacia Pinheiro, Alvin H. Schmaier

**Affiliations:** ^1^ Department of Medicine, Hematology and Oncology, University Hospitals Cleveland Medical Center, Case Western Reserve University, Cleveland, OH, United States; ^2^ Instituto de Biofísica Carlos Chagas Filho, Universidade Federal do Rio de Janeiro, Rio de Janeiro, RJ, Brazil; ^3^ Center for Global Health and Diseases, Department of Pathology, Case Western Reserve University, Cleveland, OH, United States

**Keywords:** cerebral malaria, bradykinin, factor XII, prekallikrein, high molecule weight kininogen, bradykinin B2 and B1 receptors

## Abstract

Malaria is a parasitic disease of global health significance and a leading cause of death in children living in endemic regions. Although various Plasmodium species are responsible for the disease, *Plasmodium falciparum* infection accounts for most severe cases of the disease in humans. The mechanisms of cerebral malaria pathogenesis have been studied extensively in humans and animal malaria models; however, it is far from being fully understood. Recent discoveries indicate a potential role of bradykinin and the kallikrein kinin system in the pathogenesis of cerebral malaria. The aim of this review is to highlight how bradykinin is formed in cerebral malaria and how it may impact cerebral blood-brain barrier function. Areas of interest in this context include Plasmodium parasite enzymes that directly generate bradykinin from plasma protein precursors, cytoadhesion of *P. falciparum* infected red blood cells to brain endothelial cells, and endothelial cell blood-brain barrier disruption.

## Introduction

Malaria continues to be a major cause of morbidity and mortality in tropical and sub-tropical areas of the world. Life-threatening clinical malaria syndromes – severe anemia, respiratory distress accompanied by metabolic acidosis, cerebral malaria (CM) and combinations therein – primarily affect children and pregnant women with *Plasmodium falciparum* (*Pf*) infection. In 2021 there was an estimated 619,000 malaria deaths world-wide; 593,000 of these deaths occurred in sub-Saharan Africa (WHO, 2022). Children younger than 3-5 years with little prior exposure to *Pf* blood stage infection and minimal naturally acquired immunity to clinical malaria are the major age group at risk of death from CM. Most deaths from *Plasmodium falciparum* (*Pf*) infection are due to cerebral malaria.

Cerebral malaria is a neurological complication caused by *Pf* blood stage infection. The World Health Organization has defined 3 clinical criteria for the diagnosis of CM: i) a peripheral blood smear or rapid diagnostic test positive for asexual stage *Pf* parasites; ii) exclusion of any other etiology of neurologic dysfunction; and iii) coma and seizures (W. H. O, 2012). Magnetic resonance imaging studies of children and adults with CM indicate that vasogenic edema resulting from breakdown of the blood-brain barrier (BBB) underlies progressive brain swelling that can lead to fatal brainstem herniation (Seydel et al., 2015; Mohanty et al., 2017). The exact pathophysiological mechanisms underlying cerebral edema with hemorrhage are not known. Systemic inflammation, activation of coagulation and the cytokine cascade, neutrophil degranulation, and cytoadherence of *Pf* infected red blood cells (iRBCs) to brain vascular endothelium are collectively thought to initiate the breakdown of the BBB (Moxon et al., 2009; Moxon et al., 2020; Patel et al., 2020; Guenther et al., 2021). Fundoscopic examination of the eye has revealed that distinctive pathologic features of retinal blood vessels, e.g., macular and peripheral whitening due to ischemia associated with iRBC cytoadhesion, are associated with high risk of fatal CM relative to CM cases without these retinal changes (Beare, 2023; Wilson et al., 2023). Seminal studies conducted in Africa and Southeast Asia indicate that prompt treatment with parenteral artesunate reduces the death rate of CM relative to quinine, the previous standard of care (Dondorp et al., 2010). Nevertheless, 10 percent of children with severe malaria of any clinical phenotype die despite appropriate treatment (Gething et al., 2016; Maitland, 2016).

Children who recover from CM and other severe malaria syndromes may experience long-term neurocognitive and motor deficits (John et al., 2008; Idro et al., 2016). Persistent elevation of plasma biomarkers of inflammation, such as C-reactive protein, following clinical recovery have been associated with these deficits (Conroy et al., 2021). However, there is minimal understanding of the etiology and prevention of long-term neurologic sequelae in children who survive severe malaria.

The proximate molecular pathways underlying progressive brain edema in CM are not known. Pathologic examination of brains of children with fatal CM shows extensive intravascular sequestration of iRBCs with vascular thrombosis, perivascular hemorrhage, and infiltration of adjacent brain parenchyma with monocyte/macrophages, hemozoin (a parasite byproduct of hemoglobin degradation), and CD8^+^ T cells (Jensen et al., 2020; Moxon et al., 2020; Riggle et al., 2020). Immunostaining studies of brain slices indicate that platelet and neutrophil activation have occurred *in vivo* (Amulic et al., 2020). Relative to children with non-life threatening uncomplicated malaria, iRBCs isolated from the blood of children with CM express variants of *Pf* erythrocyte protein-1 (PfEMP1) on the iRBC surface that bind to intercellular adhesion molecule 1 (ICAM-1) and endothelial protein C receptor (EPCR) on the brain endothelial cell (EC) luminal surface (Kessler et al., 2017; Sahu et al., 2021; Ramachandran and Sharma, 2022). Sequestration of iRBCs in cerebral microvessels produces mechanical obstruction to blood flow and metabolic disturbances in the EC and adjacent brain tissue. Infected RBC binding triggers EC signaling pathways that lead to reorganization of endothelium tight junction complexes and leakiness of the BBB (van der Heyde et al., 2006; Wassmer and Grau, 2017). Disruption of BBB and plasma leakage are hallmark features in CM pathology, as demonstrated in murine experimental cerebral malaria (ECM) by measuring extravasation of impermeable dyes into the brains of infected mice where local hemorrhage is observed (Renia et al., 2012).

In this review, we ask the question, what is the evidence for bradykinin, a biologic peptide with vasogenic properties, as an etiologic agent in the pathogenesis of cerebral malaria? This review is a summary of human, animal, and *in vitro* data addressing this question. Our focus is the generation of bradykinin in CM and its possible vascular effects on infected patients.

## Bradykinin formation in the intravascular compartment

### Bradykinin generation

**Figure 1:**
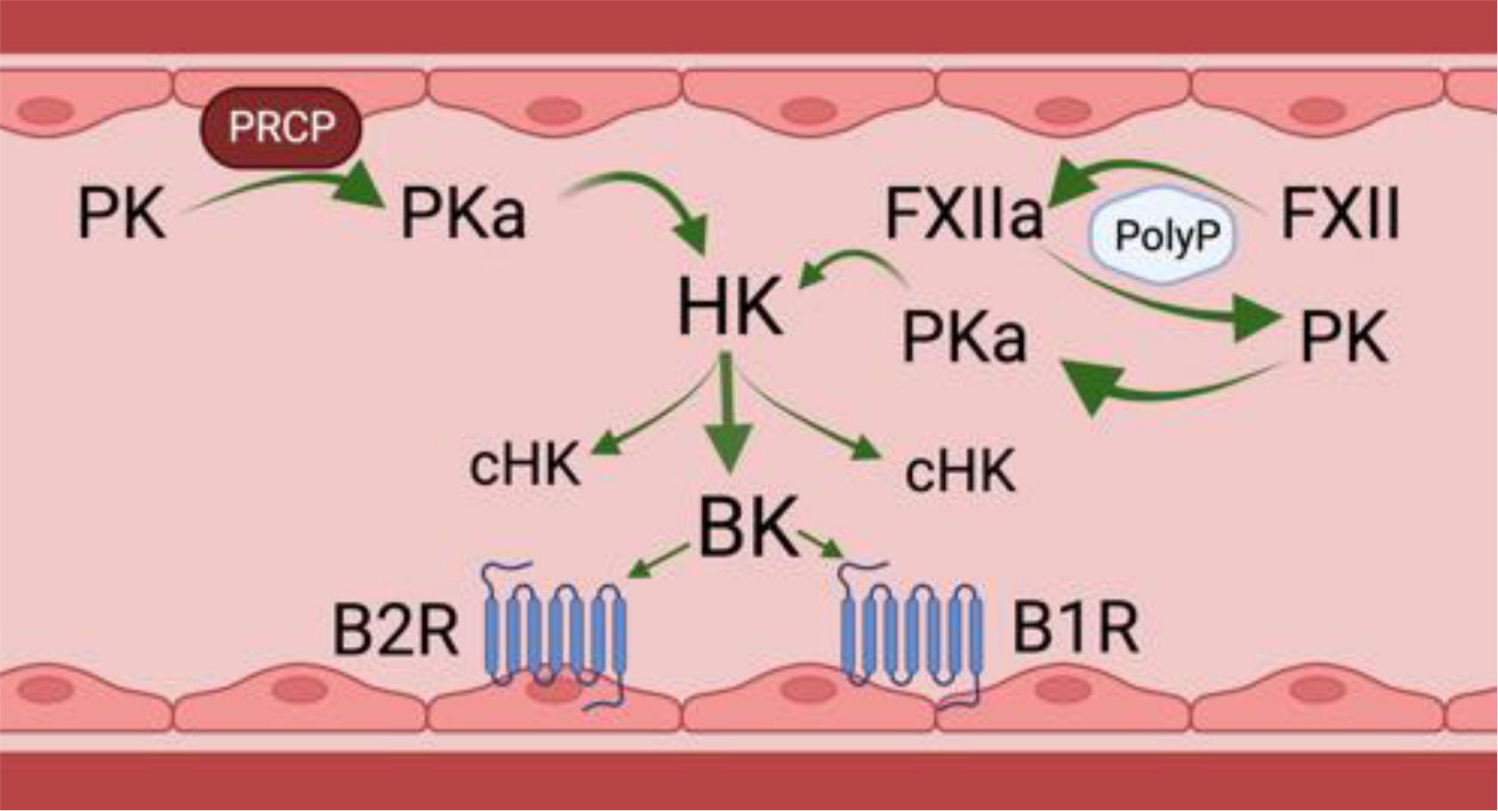
Physiologic bradykinin forming pathways in the intravascular compartment. PK, prekallikrein; PKa, plasma kallikrein; PRCP, prolylcarboxypeptidase; FXII, factor XII; FXIIa, activated factor XII; HK, high molecular weight kininogen; cHK, cleaved HK or bradykinin-free HK; BK, bradykinin; B2R, bradykinin B2; B1R, bradykinin B1 receptor. There are two physiologic mechanisms for BK formation in the intravascular compartment. The first is on the left of the figure. It shows that on a negatively activated surface, e.g., PolyP, factor XII autoactivates into an enzyme FXIIa. FXIIa then activates zymogen PK to plasma PKa). A second mechanism to form PKa is on the right. PK bound to endothelial cells by HK is proteolyzed by a membrane expressed PRCP. PKa formed by any mechanism cleaves the HK it is bound to, liberating BK and leaving a residual bradykinin-free HK or cHK. Formed BK binds to its endothelial cell constitutive receptor, the B2R to stimulate endothelial cell activation. In inflammatory states, a second receptor, termed the B1R also is expressed to mediate BK’s effect on endothelial cell biology. Figure made with Biorender.

Bradykinin (BK) is a 9 amino acid peptide (RPPGFSPFR) liberated by cleavage of high or low molecular weight kininogen (HK and LK) by plasma kallikrein (PKa), tissue kallikrein, and/or activated factor XII (FXIIa). The *in vivo* half-life of BK is less than 1 minute as various plasma proteases rapidly degrade the peptide (Cicardi and Zuraw, 2018; Pinheiro et al., 2022). Related peptides with similar biological effects on ECs, i.e., the capacity to increase vascular permeability and local angioedema, include Lys BK, a decapeptide with an amino acid sequence identical to BK with lysine at position 10 formed by tissue kallikreins and desArg^9^BK, an octapeptide with an amino acid sequence identical to BK with Arg lacking at position 9 formed by several BK carboxypeptidases (Pinheiro et al., 2022). Lys BK is liberated from LK by tissue kallikrein. In general, tissue kallikreins do not participate in cleaving HK within the intravascular compartment. PKa and FXIIa of the plasma contact activation system are present as enzymatically inactive zymogens [denoted respectively as prekallikrein (PK) and factor XII (FXII)]. Negatively charged biological molecules in plasma, such as polyphosphates, cell lysates, collagen, lipids, aggregated proteins and artificial products, support FXII auto-activation to FXIIa (Colman and Schmaier, 1997) (**Figure 1**, on the right). Small amounts of FXIIa catalytically hydrolyze plasma PK to PKa which, in turn, produces more FXIIa in reciprocal activation leading to amplification of activation. Both PKa and FXIIa cleave HK or LK between a Lys-Arg at the N-terminus of BK and Arg-Ser at the C-terminus to liberate the intact peptide (Schmaier, 2016). HK also binds to EC, platelets, and neutrophils to serve as a receptor for PK (Gustafson et al., 1986; Schmaier et al., 1988).

In addition to contact activation, there is another pathway that leads to PK activation on the vessel wall, Endothelial cell prolylcarboxypeptidase (PRCP) is a serine protease that also hydrolyzes PK to PKa when bound to HK on the cells to liberate BK (Pinheiro et al., 2022) (**Figure 1**, on the left). This pathway is independent of FXII. On ECs the degree of PK activation is regulated inversely by the ambient concentration of C1 inhibitor (C1INH) and directly by PRCP (Kaplan and Joseph, 2017; Merkulova et al., 2023). FXII binds ECs, but in the absence of PK and HK, it does not autoactivate to FXIIa. Even in the presence of high concentrations of C1INH, the degree of inhibition of cleavage of HK and BK liberation by forming plasma kallikrein is only 50% (Kaplan and Joseph, 2017; Merkulova et al., 2023). This statement implies that some BK is always being formed on EC regardless of the C1INH concentration.

### Bradykinin degradation and binding to endothelial cell receptors

Angiotensin converting enzyme (ACE) is the major BK degrading enzyme in the intravascular compartment. In total, there are 13 plasma and tissue BK peptidases (Pinheiro et al., 2022). Carboxypeptidases degrade BK and Lys-BK to DesArg^9^BK and LysDesArg^9^BK, respectively, and are ligands for the BK G-protein coupled B2 receptor (B2R) and B1 receptor (B1R), respectively (**Figure 1**). The B2R is the constitutive receptor in the intravascular compartment. The B1R is expressed in inflammatory states or as a response to injury. BK binding to B2R on the EC surface stimulates the release of nitric oxide and prostacyclin, followed by loss of BBB function, increased vascular permeability, and vasodilation due to decreased smooth muscle cell tone. It is not known if the BK signaling pathway for loss of BBB function is the same for vasodilation.

### Bradykinin in non-malarial diseases

**Figure 2:**
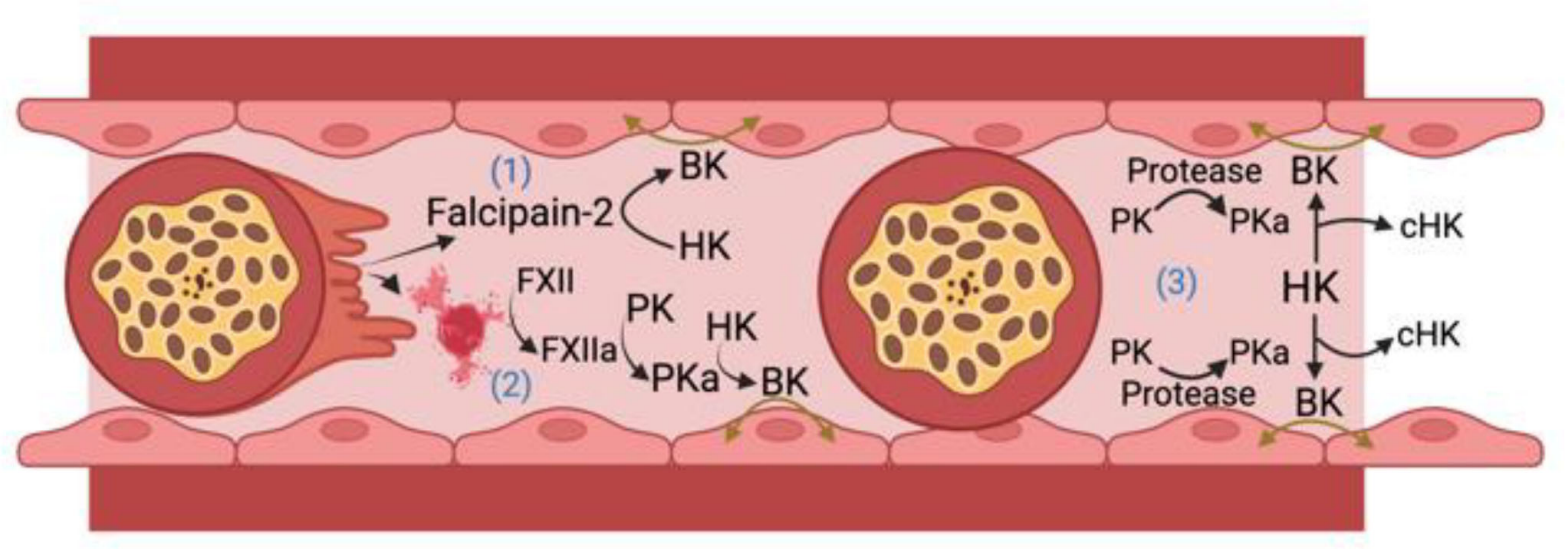
*Bradykinin (BK) formation in cerebral malaria.* HK, high molecular weight kininogen; PK, plasma prekallikrein; PKa, plasma kallikrein; FXII, factor XII; FXIIa, activated FXII; cHK, cleaved (bradykinin free) high molecular weight kininogen. There are 3 ways iRBCs may lead to BK formation. 1) Falcipain-2 itself released from *Plasmodium falciparum* cleaves HK to liberate BK; 2) The ruptured membranes or microparticles/exosomes from *P. falciparum* infected RBCs or parasites themselves support FXII autoactivation leading to PKa formation that liberates BK from HK; 3) Occluding *P. falciparum* infected red blood cells upregulate an endothelial cell PK activator (Protease) that leads to more PKa activation with cleavage of HK liberating BK to produce edema in the vessel wall. Figure made with Biorender.

The archetype contact activation system disease state is hereditary angioedema, an inflammatory disorder manifested by localized edema in skin, soft tissue, bowel, and the larynx. This condition has been associated with defects or deficiency of C1 inhibitor, FXII, plasminogen, angiopoietin 1, HK, heparan sulfate, and myoferlin (Schmaier et al., 1986; Kaplan and Joseph, 2017; Schmaier, 2019; Kaplan et al., 2022). Since BK formation leads to tissue edema with associated microvessel thrombosis, we hypothesize that proteins of the plasma contact activation system and BK participate in CM pathogenesis by mediating breakdown of the BBB. In addition, we propose that BK itself may impact malaria parasite growth and transmission to mosquito vectors. The following is a presentation of what is known about the participation of FXII and the KKS in BK formation in malaria. We will describe published evidence suggesting that: 1) BK is generated during malaria infection; 2) BK is a potential causal agent of vascular leakage in humans and experimental animals with severe malaria, including CM; 3) A cysteine protease expressed by Pf parasites directly generates BK from HK; 4) BK has direct anti-plasmodium activity. A schematic of potential pathways by which BK may contribute to CM pathogenesis is shown in **Figure 2**.

## Malaria infection induces bradykinin formation and vascular permeability *in vivo* in humans, primates, and mice.

### Human studies

Observational studies of human malaria conducted in the first half of 20^th^ century suggested that increased vascular permeability and capillary leakage contribute to the pathogenesis of severe malaria. Estimated changes in intravascular versus extravascular plasma distribution in patients undergoing malaria-induced “fever therapy” for the treatment of neurosyphilis were among the first to suggest that Pf blood stage infection leads to vascular leakiness (O'Leary, 1928; Wagner-Jauregg and Bruetsch, 1946; Verhave, 2013; Kraft et al., 2019). Notably, this form of therapy for neurosyphilis, used in the pre-antibiotic era before the discovery and widespread availability of penicillin, did not undergo rigorous randomized clinical trials. From today’s vantage point, these historical therapies appear neither safe nor ethical.

In a report describing 9 malaria naïve neurosyphilis patients inoculated intravenously with *Pf* or *P. vivax* iRBCs, one of the four patients infected with *Pf* developed hypotension and decreased consciousness concomitant with high parasitemia and decreased intravascular plasma volume measured by dilution of Evans blue dye relative to the baseline value before iRBC infection (Feldman and Murphy, 1945). Recovery ensued two to three days later following transfusion of whole blood and plasma. The authors suggested that vascular leakage was a component of the acute decrease in plasma volume. In contrast, studies of American soldiers with uncomplicated non-life threatening malaria had no evidence of plasma vascular leakage measured by radio-iodinated serum albumin dilution (Malloy et al., 1967). More recently, Davis et al. (1992) measured vascular endothelium permeability by trans-capillary escape of radiolabeled albumin and the urinary albumin/creatinine ratio in adult Thai patients with severe malaria and patients with uncomplicated malaria. The former two measures of capillary leakage were higher in patients with severe malaria than patients with uncomplicated malaria. Severe malaria patients also had significantly greater trans-capillary escape of radio-labeled albumin than those with uncomplicated malaria. Another study of 22 Thai adults with severe malaria, including 6 with CM, reported that FXII and PK levels were significantly decreased at presentation, consistent with prior activation of the intrinsic pathway of coagulation (Clemens et al., 1994). The C1 inhibitor activity/antigen ratio, a biomarker for prior contact activation, was also decreased.

### Primate investigations

With respect to primate malaria, a series of studies conducted in the mid to late 1960s showed that BK-like activity measured by increased skin vascular permeability in guinea pigs was increased, and the BK precursor protein, bradykininogen (now referred to as HK), decreased in plasma of rhesus monkeys (*Macaca mulatta*) infected with *P. knowlesi* relative to uninfected controls (Tella and Maegraith, 1966; Maegraith and Tella, 1968; Maegraith and Onabanjo, 1969a; Maegraith and Onabanjo, 1969b; Maegraith and Onabanjo, 1970). In 1969, Colman et al. (Colman et al., 1969) and Maegraith and Onabanjo (1969a) showed that the kininogenase (now referred to as plasma PK) increased permeability in skin capillaries of guinea pigs. Onabanjo and Maegraith subsequently observed that brain capillaries of *P. knowlesi* infected monkeys occluded by iRBCs had elevated plasma PK, increased EC permeability, and vasodilation (Onabanjo and Maegraith, 1970a). Injection of plasma PKa fractions containing pontamine blue dye into the brain increased EC permeability along with lymphocyte and eosinophil infiltration and RBC diapedesis (Onabanjo and Maegraith, 1970b). Increased plasma PKa also was associated with a fall in plasma bradykininogen (HK) in infected animals (Onabanjo and Maegraith, 1970c). Finally, severe hypotension in rhesus monkeys infected with *P. knowlesi* was found to be accompanied by PK activation, cleavage of HK, and high BK levels (Skirrow et al., 1964).

### Rodent studies

The above observations in primate malaria are similar to those reported in studies of mice with experimental CM following infection with *Plasmodium berghei* (NK65 strain) (Ohtomo and Katori, 1972). Plasma HK levels were markedly reduced and kinin levels increased concomitant with progressive severity of neurologic dysfunction. Further, treatment of *P. berghei* ANKA infected mice with captopril, an ACE inhibitor, increased BK levels and directly modulated CD8^+^ T cell responses (Silva-Filho et al., 2013; Silva-Filho et al., 2017). These combined studies indicate that malaria infection in rodents and primates was associated with plasma proteins that contribute to the generation of BK.

## Effect of *Plasmodium*-infected red blood cells on bradykinin formation and human brain endothelial cell biology—in vitro studies

*Plasmodium falciparum* iRBCs internalize plasma HK and actively process it through cysteine proteases to liberate BK and its analogs to the extracellular space (Bagnaresi et al., 2012). This process may contribute to activation of the kallikrein-kinin system in ECs. Based on this precedent, investigations examined whether kinins generated in the conditioned medium of *Plasmodium falciparum* could modulate iRBC cytoadhesion as well as barrier function (Silva et al., 2019). The conditioned media of *Pf* cultures stimulated B2R and B1R to increase iRBC cytoadhesion to brain microvascular endothelial cells. These activities were blocked by antagonists to B2R (HOE-140) and B1R (DALBK). The same results were observed when cells were treated with BK alone. Bradykinin or conditioned medium of Pf cultures stimulation of brain EC monolayers impaired tight junctions with loss of ZO-1 and β-catenin expression on cell surface, resulting in changes in EC function that allowed for increased permeability of bovine serum albumin tracer (Silva et al., 2019).

## High molecular weight kininogen is a substrate of falcipain-2 with bradykinin formation

Kininogens such as HK have been known to be substrates and inhibitors of cysteine proteases for over 40 years. We showed that HK inhibits platelet calpain 2 with high affinity, and molar excess calpain 2 destroys HK (Schmaier et al., 1986; Bradford et al., 1990). Further, in stochiometric ratios (1:1) of calpain:HK, BK is liberated from HK (Higashiyama et al., 1986). These data serve as a guide to how falcipains, a *Pf* cysteine protease, cleaved HK to liberate BK. It has been reported that the *Pf* aminopeptidase P in food vacuoles hydrolyzes BK (Ragheb et al., 2009). *Plasmodium chabaudi* and *Pf* iRBCs also internalize and process plasma HK that subsequently release the vasoactive kinins, Lys-BK, BK, and desArg^9^BK (Bagnaresi et al., 2012). Furthermore, recombinant falcipain-2 and -3 proteolyze HK to liberate functional kinins that induced ileum contraction and activated the B2R and B1R as measured by calcium mobilization. These activities were blocked with antagonists to the B2R (HOE140) and B1R [DesArg^9^(Leu8)] and, independently, E64 protease. In other studies, both falcipain-2 and falcipain-3 generated BK (Cotrin et al., 2013). Both enzymes generated Met-Lys-BK, Lys-BK, and BK from the substrate Abz-MISLMKRPPGFSPFRSSRI-NH_2_. BK formed by any means stimulates nitric oxide production, induces vasodilatation, activates EC, enhances microvascular permeability, and modulates cellular metabolism.

## Influence of bradykinin on *Plasmodium* growth

The role of BK in malaria depends on its local or systemic production, the correlate BK receptor to which it binds and signals, and *Plasmodium* species involved. Bradykinin has been reported to have anti-plasmodial activity against *P. gallinaceum*, a malarial strain that infects chickens (Silva et al., 2015).

In other studies, the addition of BK to *P. falciparum* schizont cultures inhibited parasite growth similarly to captopril, an ACE inhibitor (Saraiva et al., 2011; Silva et al., 2016). ACE connects the kallikrein/kinin system (KKS) to the renin-angiotensin (RAS) and its inhibition leads to increase in both BK and Ang- (Moxon et al., 2009; W. H. O, 2012; Seydel et al., 2015; Mohanty et al., 2017; Santos et al., 2018; Moxon et al., 2020; Guenther et al., 2021; WHO, 2022) levels. In fact, the effect of BK was prevented by HOE-140 or A779, blockers of B2 or MAS receptors, respectively. Also, BK-induced inhibition of PKA activity in erythrocytes was sensitive to both pharmacological treatments, suggesting a coordinated effect between RAS and KKS. Co-immunoprecipitation assays demonstrated a possible association between B2R and Mas in the RBC membrane. B2R and Mas heterodimers have been demonstrated in the EC surface membrane as well. However, if this mechanism occurs during malaria infection remains to be determined (Abadir et al., 2006).

## Conclusions

Published data reviewed here suggest that BK may be generated and contribute to the pathogenesis of vasogenic edema in CM in several ways (**Figure 2**). Falcipain-2 from the parasite itself cleaves HK and liberates BK. Further, malaria iRBCs and their microparticles released into the circulation provide negatively charged surfaces allowing for FXII autoactivation with activation of PK to plasma PKa leading to cleavage of HK and liberation of BK. Lastly, iRBC occlusion of microvessels in the brain could perturb the EC membrane and allow for vessel wall associated proteases such as prolylcarboxypeptidase to activate PK to produce plasma PKa to cleave HK with BK liberation that also contributes to reciprocal activation of FXII. Future investigations need to show causality of the proteins of the factor XII and kallikrein/kinin system in CM. These approaches should be done with global and selective gene deleted mice along with the use of precise pharmacologic agents already developed to treat diseases like hereditary angioedema (Abdulkarim and Craig, 2023; Wilkerson and Moellman, 2023). More in depth studies of the role(s) of contact activation and kallikrein-kinin system in CM may provide justification to test these drugs as adjunctive therapeutics to anti-malarial drugs. Finally, future studies of cerebral malaria, other severe malaria syndromes, and uncomplicated malaria that should include measurements of BK levels and activation of the kallikrein-kinin system before and after treatment with antimalarial drugs.

## Author contributions

All authors contributed to the manuscript. AP wrote the first draft. The other authors worked together to complete the manuscript. All authors have seen the final version of the manuscript.
